# CgMyD88s Serves as an Innate Immune System Plug During Ostreid Herpesvirus 1 Infection in the Pacific Oyster (*Crassostrea gigas*)

**DOI:** 10.3389/fimmu.2020.01247

**Published:** 2020-07-14

**Authors:** Xueying Tang, Baoyu Huang, Siheng Lin, Wei Wang, Guofan Zhang, Li Li

**Affiliations:** ^1^CAS Key Laboratory of Experimental Marine Biology, Institute of Oceanology, Chinese Academy of Sciences, Qingdao, China; ^2^Laboratory for Marine Biology and Biotechnology, Qingdao National Laboratory for Marine Science and Technology, Qingdao, China; ^3^Department of Biological Sciences and Biotechnology, Minnan Normal University, Zhangzhou, China; ^4^Center for Ocean Mega-Science, Chinese Academy of Sciences, Qingdao, China; ^5^National & Local Joint Engineering Laboratory of Ecological Mariculture, Qingdao, China; ^6^Laboratory for Marine Fisheries Science and Food Production Processes, Qingdao National Laboratory for Marine Science and Technology, Qingdao, China

**Keywords:** innate immunity, OsHV-1 μVar, *Crassostrea gigas*, TLR, MyD88

## Abstract

Ostreid herpesvirus-1 microvariant (OsHV-1 μVar) is considered a major infectious microbe that can reduce the survival of natural or cultured oysters in summer. Because they lack an adaptive immune system, oysters are dependent on their innate immune systems to fight pathogens. The duplication and functional divergence of innate immune genes in the oyster have been studied, but the contribution of molecular mechanisms underlying innate immunity remains to be defined. Here, we identified the interacting proteins associated with *Crassostrea gigas* Toll-like receptors (CgTLR) using a yeast two-hybrid (Y2H) screening system. A total of eight proteins were identified that could interact with CgTLR. Three of these appeared at least four times in the screening and were related to MyD88. Two genes encoding these MyD88-like proteins, CgMyD88-1 and CgMyD88-2, possessed typical death and TIR domains. The third gene encoding an MyD88-like protein possessed only a TIR domain, and we named it CgMyD88s. CgMyD88s interacted only with CgTLR, but not CgMyD88-1 or CgMyD88-2. Both CgMyD88-1 and CgMyD88-2 mRNAs were upregulated after OsHV-1 μVar infection, whereas the expression of CgMyD88s decreased. When overexpressed in HEK293T cells, CgMyD88-1 and CgMyD88-2 activated an NF-κB reporter, whereas CgMyD88s impaired activation induced by CgMyD88-1 or CgMyD88-2. Intriguingly, the silencing of CgMyD88s using double-stranded RNA (dsRNA)-mediated RNA interference increased the expression of CgMyD88-1 and CgMyD88-2. Taken together, our results revealed that CgMyD88-1, CgMyD88-2, and CgMyD88s may all participate in the TLR-mediated innate immune pathway and that CgMyD88s served as a plug to avoid oysters from excessive inflammatory response during OsHV-1 μVar infections.

## Introduction

Bivalves include clams, mussels, scallops, and oysters, and some of these, such as cultured Pacific oyster (*Crassostrea gigas*), are of great economic importance. The Pacific oyster, originally found only in East Asia, has been successfully introduced into many countries and has become a major aquaculture species worldwide, because *C. gigas* can tolerate harsh and dynamically changing environments (1, 2). However, most natural and many cultured oyster populations experience mass mortality events, especially in summer (3). Summer oyster mortality is the consequence of complex interactions between the hosts, environment, and pathogens (4–6). Pathogens, especially ostreid herpesvirus 1 (OsHV-1), infect bivalve species in the aquaculture industry (7–10). An OsHV-1 microvariant, μVar, appeared during the summer of 2008 in France and now seems to be the dominant herpesvirus that infects these oysters (8, 11). Because oysters lack an adaptive immune system, innate immunity serves as the bivalve defense system, playing a critical role in responding to infections (12–14). Innate immunity relies on recognition of conserved pathogen-associated molecular patterns (PAMPs) present in microbes by pattern recognition receptors (PRRs) in the hosts (15). Upon PAMP recognition, hosts initiate intracellular signaling, which uses adaptors, kinases, and transcription factors to trigger proinflammatory and antimicrobial effectors (16).

Toll-like receptor (TLR) signaling is one of the most important pathways for host immune responses against pathogen invasion (17). Myeloid differentiation factor 88 (MyD88) is a universal adaptor that is recruited to TLRs when these receptors are activated to transduce signals to downstream molecules (18). MyD88 is also considered the most important adaptor in bivalve species (19). In a previous study, annotation of the Pacific oyster genome revealed large-scale duplication and divergence of the TLR family, with 83 TLR genes and 10 MyD88-like genes in this species (20). Zhang et al. then showed that duplicated genes in TLR signaling pathways were responsive to different pathogens, as well as environmental stress (21). Although the duplication and expansion of genes in TLR signaling pathways have been established, further investigation of the signaling and regulatory networks that mediate immunity in this species to gain a better understanding of how those diverged molecules cooperate or compete with each other to protect the host from infections is needed.

The first oyster TLR, which is functionally involved in defense against bacteria, was identified before the oyster genome was published (22). Subsequently, four more TLRs in oysters were found to respond to multiple PAMP challenges and to constitutively activate the NF-κB responsive reporter (23). In addition, two MyD88 family members were upregulated in hemocytes after OsHV-1 challenge (24). Here, we choose the vertebrate-type TLR, which is highly expressed during OsHV-1 outbreaks based on viral transcriptome analysis (unpublished) and identified TLR-interacting proteins using a Y2H screening system. Our results show that CgMyD88s, a novel MyD88-like protein, competes with normal MyD88 to initiate TLR-mediated innate immunity.

## Materials and Methods

### Oyster Collection and Treatment

Adult oysters with a shell length of 7.05 ± 0.7 cm were obtained from aquaculture areas of Jiaonan in Qingdao, Shandong province, China. All of the oysters were allowed to acclimate to laboratory conditions at 18 ± 1°C with daily filtered seawater changes and feeding once daily with marine algae (*Spirulina platensis*) for 1 week. In a preliminary experiment, 10 oysters were randomly selected to test for the virus to make sure the oysters were free from OsHV-1. Before the stress experiments, oysters were administered anesthesia by exposing them to 50 g/L MgCl_2_·6H_2_O liquid for one night to open their shells. For OsHV-1 μVar infection, a viral homogenate was prepared by thoroughly mincing tissues with a high viral load in phosphate-buffered saline (PBS) and then passing the resulting suspension through 20-, 5-, 0.45-, and 0.22-μm filters (4, 25). The viral load in the homogenate was 8.4 × 10^4^ viral copy numbers measured by quantitative real-time PCR using C9/C10 primers. One hundred microliters of homogenate was injected into the adductor muscles of each oyster in the experimental group. Oysters in the control group were injected with 100 μl PBS. Each group contained at least 40 oysters. After injection, hemocytes were collected from five oysters per group at 0, 3, 6, 12, 24, 48, and 72 h. The oyster hemocytes were collected from the hemolymph, which was obtained from the pericardial cavity using 1-ml sterile syringes and immediately centrifuged at 1,000 × g for 10 min at 4°C and then stored at −80°C until use.

### RNA Extraction and cDNA Synthesis

TRIzol reagent was used to isolate total RNA from hemocytes according to the manufacturer's instructions (Invitrogen, USA). One microgram of total RNA was used to synthesize first-strand cDNA with a PrimeScript RT Reagent Kit (TaKaRa, China). The cDNA was then used as a template to construct a recombinant plasmid or to analyze mRNA expression.

### Construction, Autoactivation, and Toxicity Detection of the Bait Protein Expression Plasmid CgTLR-TIR-BD

A CgTLR gene sequence was obtained from GenBank (GenBank accession number: KC700619) and used to design original 16-bp primers. The primers used to amplify the toll/interleukin-1 receptor (TIR) domain of CgTLR were CgTLR-TIR-BD-F and CgTLR-TIR-BD-R. All of the primers used in this study are listed in Table S1. The empty pGBK-T7 plasmid was digested by both *Bam*HI and *Eco*RI and then fused with a purified CgTLR-TIR-BD PCR product using a Ligation-Free Cloning System according to the manufacturer's instructions (ABM, Inc., Ontario, Canada). The recombinant plasmid was transformed into Trans-T1 Phage Resistant Chemically Competent Cells (TransGen, Beijing, China) and extracted with an EndoFree Mini Plasmid Kit II (Tiangen, Beijing, China).

The correctly constructed CgTLR-TIR-BD plasmid was transformed into yeast strain Y2HGold, as well as empty pGBK-T7 as a control, according to the instructions of the Yeastmaker™ Yeast Transformation System 2 (Clontech). We then used 100 μl of a 1/100 dilution to coat the selective media SD/–Trp, SD/–Trp/X-α-Gal, and SD/–Trp/AbA/X-α-Gal. The toxicity of the bait protein expression plasmid CgTLR-TIR-BD was determined based on growth of bacterial colonies on the plates.

### CgTLR Interaction Screening and Bioinformatics Analysis

The bait yeast strain Y2HGold was inoculated with the plasmid CgTLR-TIR-BD into 3 ml YPDA liquid medium at 30°C and 250 rpm for 8–12 h. Five microliters of selective liquid medium was added to 50 ml fresh YPDA liquid medium to reach an OD_600_ of 0.4–0.5 at 30°C and 250 rpm. The supernatant was discarded after centrifugation for 5 min at 700 × g. The cells were then resuspended in 1.5 ml 1.1 × TE/LiAc, incubated on ice for 15 min, transferred to 1.5-ml microcentrifuge tubes, and briefly centrifuged (10,000 × g for 15 s). The supernatant was discarded, and the cells were resuspended in 600 μl fresh 1.1 × TE/LiAc and then stored on ice until use. The homogenizable yeast cDNA library of oysters was constructed by TaKaRa Bioindustry.

A transformation mix for library transformation reactions was prepared as follows: (1) 10 μg of yeast cDNA from an oyster and 20 μl of carrier DNA (10 mg/ml, degenerated) were added to 2.5 ml of PEG/TE/LiAc mixture in a sterile 5-ml tube. (2) This was added to 600 μl of freshly prepared competent yeast cells, and the mixture was vortexed. The tube was then placed at 30°C for 45 min with gentle shaking, 160 μl of DMSO was added following transfer of the tube to a water bath at 42°C, and this mixture was incubated for 20 min. After 20 min, the tube was centrifuged at 2,000 × g for 5 min at 25°C. The supernatant was discarded, and the cells were resuspended in 3 ml of YPD Plus liquid medium. The tube was incubated for 90 min at 30°C and 250 rpm and then centrifuged at 2,000 × g for 5 min. After discarding the supernatant, the cells were resuspended in 10 ml PBS, and 12 μl was inoculated into 108 μl PBS. Next, 100 μl of 1/100 and 1/1,000 dilutions were used to coat SD/–Trp-Leu (double dropout, DDO) selective medium to calculate the transformation efficiency. Two hundred microliters of the remaining suspension was used to coat the selective medium SD/–Leu-Trp-His/Kan (about 50 plates as triple dropout, TDO), and the plates were incubated for at least 3–5 days at 30°C. The yeast colonies which grew on these selective TDO plates were picked up and sprayed dot to a higher-stringency SD/–Trp-Leu-Ade-His/X-α-Gal/AbA/Kan (quadruple dropout, QDO/X/A) plate. Growth of blue yeast colonies on the QDO plates was considered potential positive interactions.

Mating efficiency was checked by determining the total numbers of resulting colonies on 1/100- and 1/1,000-dilution DDO plates. Then, mating efficiency was calculated using the following formula:

$$\begin{array}{l} \text{mating efficiency}=\frac{\text{No}.\text{ of colonies }\times \text{ total volume of solution}}{\text{volume on per plate}\times \text{dilution ratio}\times \text{amount of library plasmid}}\text{cfu}/\text{μ}g. \end{array}$$

The blue yeast colonies on QDO/X/A medium were identified as positive interactions by yeast colony PCR. The inserted fragments associated with these interactions were sequenced and subjected to BLASTx analysis in GenBank.

### Cloning and Sequence Analysis

The CgMyD88s fragment was retrieved by Y2H screening, and its sequence determined by colony PCR and sequencing. Rapid amplification of cDNA ends (RACE, Invitrogen) was performed to sequence the unknown 5′ and 3′ ends of fragments. For CgMyD88s 3′ sequencing, the primers CgMyD88s-RF1/dTAP and CgMyD88s-RF2/AP were employed for primary and nested PCR, respectively. Similarly, the 5′-end sequence was obtained by nested PCR using the CgMyD88s-RR1/dGAP and CgMyD88s-RR2/AP primer pairs based on cDNA templates with a poly(C) tail added by terminal deoxynucleotidyl transferase (TdT, TaKaRa).

The open reading frame (ORF) was obtained using the ORFfinder online program (https://www.ncbi.nlm.nih.gov/orffinder/). Comparative analysis of the derived amino acid sequence was carried out in Clustal Omega (https://www.ebi.ac.uk/Tools/msa/clustalo/).

### RNA Interference and Quantitative Real-Time PCR

CgMyD88s-specific dsRNA fragments were constructed to silence the expression of CgMyD88s. Specific primers (CgMyD88s-IF and CgMyD88s-IR) with T7 promoter sequences were designed to amplify CgMyD88s (the T7 promoter sequence is underlined in Table S1). The EGFP gene (GenBank No. EU716633.1) was used as a negative control. The target fragments were amplified using Phusion High-Fidelity DNA polymerase (Thermo, Waltham, MA, USA). Subsequently, the PCR products were purified and transcribed into dsRNA using a TranscriptAid T7 High Yield Transcription Kit according to the manufacturer's instructions (Thermo, Waltham, MA, USA). CgMyD88s and EGFP dsRNA was diluted to 1 μg/μl in PBS for use. Before stimulation, 90 oysters were divided into three groups and placed under anesthesia. Each oyster in the experiment group, negative control group, and empty group was injected with 100 μl of dsRNA CgMyD88s, EGFP, and PBS, respectively. After stimulation, hemocytes were collected from four animals per group at 0, 12, 24, 48, 72, 96, and 120 h. Hemocyte cDNA was obtained as described above.

mRNA expression levels were quantified by real-time PCR using a SYBR Green Real-Time PCR Master Mix Kit (TaKaRa). β-Actin was employed as the internal control gene for cDNA normalization (26). Cycling conditions were as follows: 40 cycles of 95°C for 5 s and 60°C for 30 s. Target gene expression profiles were calculated using the 2^ΔΔ*CT*^ method normalized with β-actin (27). Data were expressed as mean and standard error of the mean. Three individuals at each time were tested, each assayed in triplicate. Statistical analysis of the normalized CT values was performed with Student's *t*-test using SPSS 23.0 program. Differences were considered significant at *p* < 0.05 (two-tailed test).

### Plasmid Construction, Cell Culture, and Transfection

The ORFs of genes studied in the following experiments were amplified using specific primers listed in Table S1. The PCR products were then ligated into the restriction enzyme cutting sites of corresponding linearized plasmids, including the *Eco*RI site of pCMV-N-Myc and pCMV-N-Flag, *Xho*l site of pGAD-T7, and *Bam*HI/*Eco*RI site of pGBK-T7. The methods were similar to those used to construct recombinant plasmid CgTLR-TIR-BD as described above. CgTLR, CgMyD88-1, CgMyD88-2, and CgMyD88s were fused with pCMV-N-Myc, and CgMyD88s was fused with pCMV-N-Flag for co-immunoprecipitation (co-IP) and dual-luciferase reporter assays.

Because no oyster cell line was available for efficient subculture, human embryonic kidney (HEK293) T cells were used express oyster proteins *in vitro* in the following experiments. HEK293 T cells were cultured in Dulbecco's Modified Eagle's Medium (Gibco, Grand Island, NY, USA) supplemented with 10% heat-inactivated fetal bovine serum (FBS) and 1 × penicillin–streptomycin solution at 37°C with 5% CO_2_, with subculture every 3 days.

To transiently transfect eukaryotic expression plasmids with oyster proteins into HEK293 T cells, Lipofectamine 3000 (Life Technologies, Carlsbad, CA, USA) was used. Flag (Sigma) and Myc (Roche) antibodies were purchased from Sigma-Aldrich (St. Louis, MO, USA) and Roche (Basel, Switzerland), respectively.

### Y2H System and Co-IP

For the Y2H, CgMyD88s-AD was transformed into the Y2HGold yeast strain (Clontech), whereas CgTLR-BD, CgMyD88-1-BD, and CgMyD88-2-BD were transformed into the Y187 yeast strain (Clontech) according to the instructions for the Yeastmaker™ Yeast Transformation System 2. The transformants were selected on Leu or Trp minimal media plates at 30°C for 3–5 days. A CgMyD88s-AD clone was hybridized with CgTLR-BD, CgMyD88-1-BD, CgMyD88-2-BD, and empty pGBK-T7 plasmid as the control according to the Matchmaker Gold Y2H System instructions (Clontech).

For co-IP, HEK293 T cells were divided and cultured in four 10-cm-diameter plates. After culturing for 24 h, four pairs of the following plasmid combinations were co-transfected into each plate: (a) CgMyD88s-Myc/CgTLR-Flag, (b) CgMyD88s-Myc/CgMyD88-1-Flag, (c) CgMyD88s-Myc/CgMyD88-2-Flag, and (d) CgMyD88s-Myc/pCMV-N-Flag as a control. Then, 24 to 36 h later, the cells were harvested using cell lysis buffer (Beyotime, Jiangsu, China). Input samples were prepared, and the remaining lysates were mixed with anti-Flag-M2 magnetic beads (Sigma-Aldrich) and incubated on a roller shaker at 4°C for 2 h. The immunocomplexes were washed twice with cold PBS and then lysis buffer. Antibody-selected proteins were eluted from the magnetic beads, as well as input samples, by boiling in SDS-PAGE loading buffer (TaKaRa) for 5 min and then analyzed by western blotting.

### Dual-Luciferase Reporter Assays

HEK293 T cells were cultured in 24-well plates until 50–60% confluency and then transfected with an NF-κB-responsive reporter vector (Beyotime), pRL-TK internal control vector (Promega), and target plasmid. The cells were washed with PBS and collected at 24 h after transfection. Transcriptional activity was measured using the Dual-Glo Luciferase Assay System (Promega, Madison, WI, USA).

### Statistical Analyses

Statistical differences were determined by unpaired two-tailed *t*-testing. *p* < 0.05 were considered significant and are marked with an asterisk (*).

## Results

### Interactions With the Protein CgTLR Were Determined by Screening Using the Y2H System

**Figure 1:**
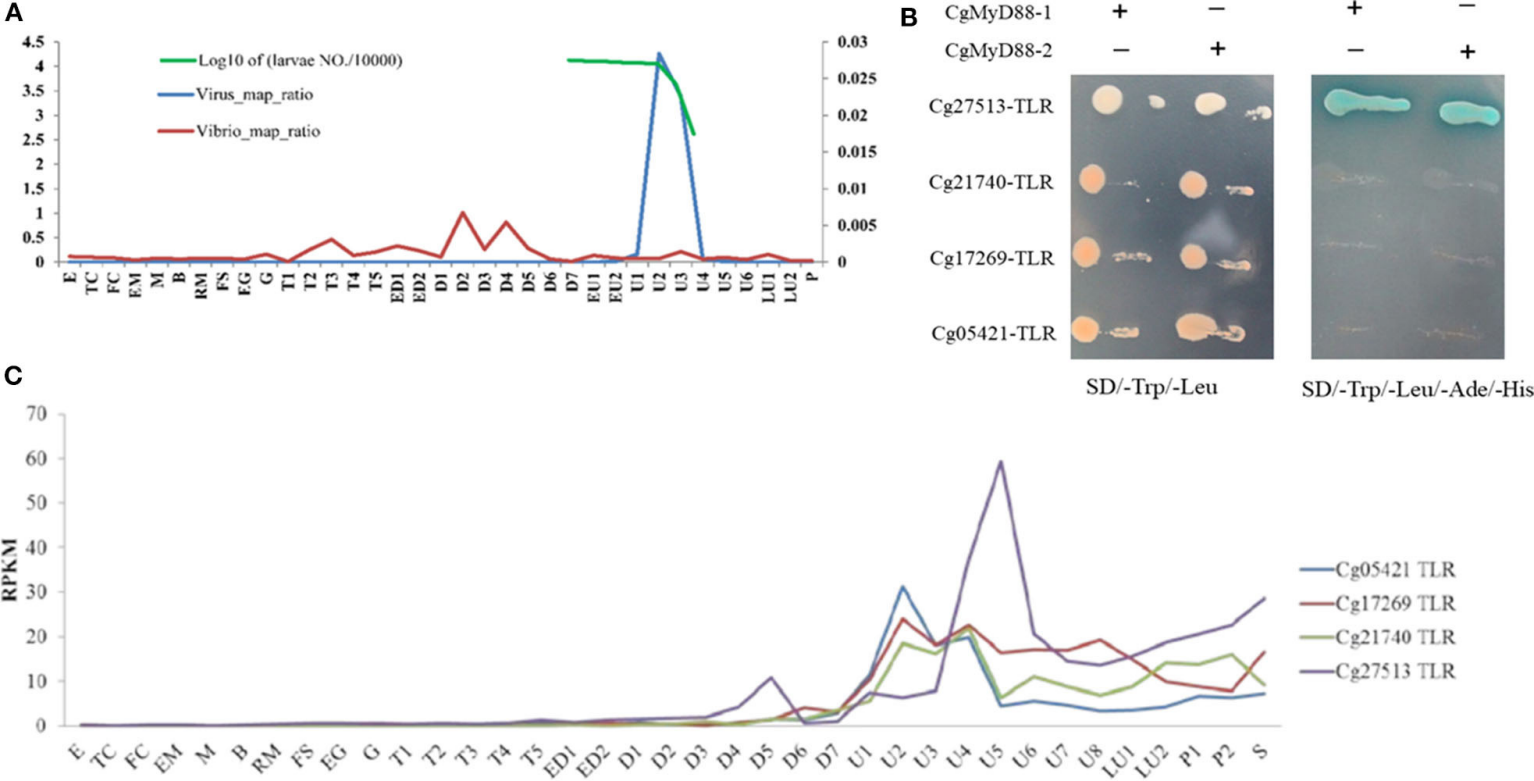
Cg27513-TLR is responsive to OsHV-1 infection and interacts with both CgMyD88-1 and CgMyD88-2. **(A)** Ratio of RNA-seq reads mapping to the OsHV-1 and *Vibrio* genomes. E, egg; TC, two cells; FC, four cells; EM, early morula; M, morula; B, blastula; RM, rotary movement; FS, free swimming; EG, early gastrula; G, gastrula; T, trochophore; ED, early D-shaped larvae; D, D-shaped larvae; EU, early umbo larva; U, umbo larva; LU, later umbo larva; P, pediveliger; and S, spat. **(B)** Expression of TLRs in oyster larvae infected with OsHV-1. **(C)** Cg27513-TLR interacted with CgMyD88-1 and CgMyD88-2 using the yeast-two-hybrid system.

As reports, the transcriptome of oyster larvae infected with OsHV-1 at different developmental stages [Figure 1A; (28)]. Only four oyster TLRs were upregulated during the outbreak period (Figure 1B). Using Y2H system, we found that just Cg27513-TLR could interact with both CgMyD88-1 and CgMyD88-2 (Figure 1C). Therefore, we chose Cg27513-TLR as CgTLR used in this study to investigate its deeper function.

**Figure 2:**
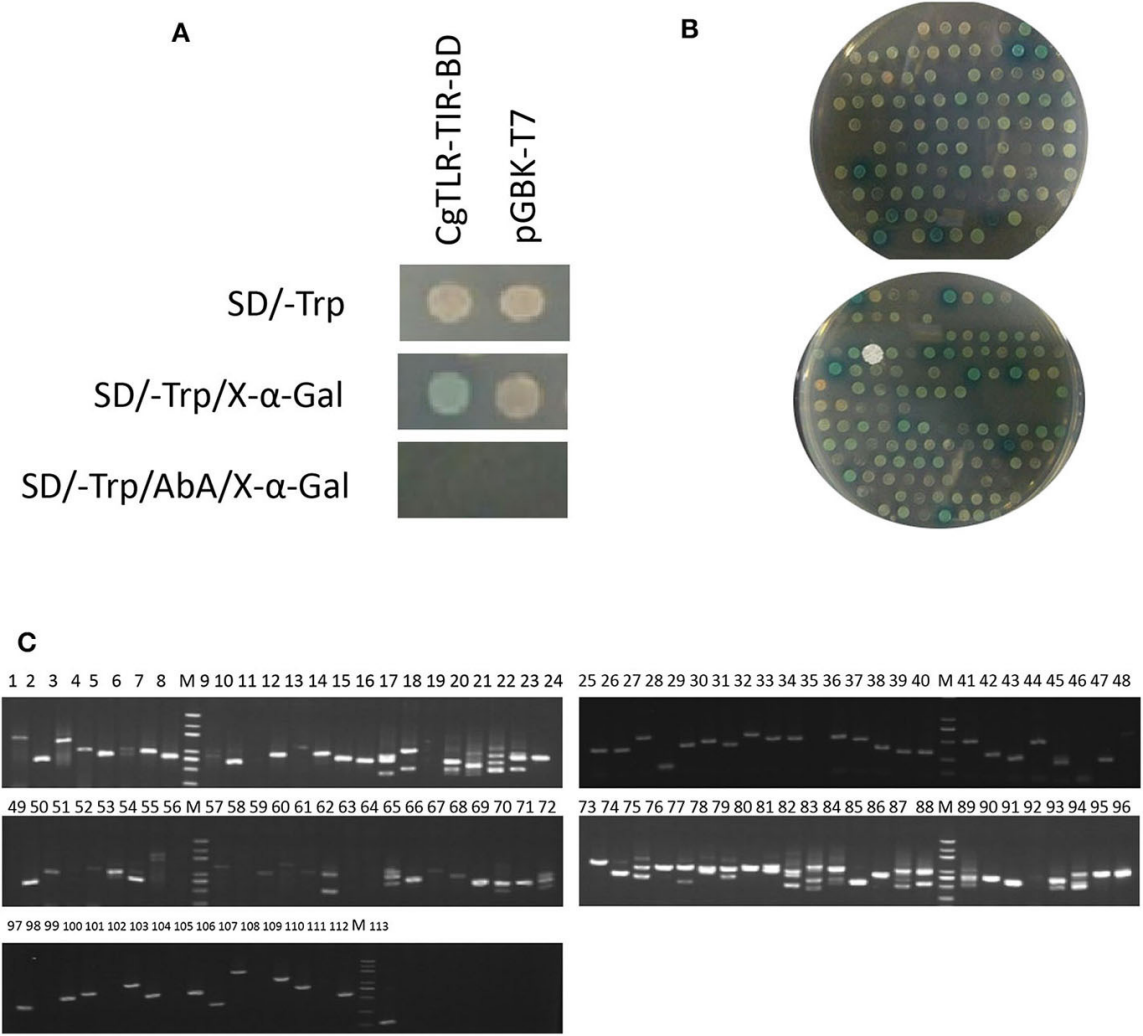
Screening of protein CgTLR interactions using the yeast two-hybrid system. **(A)** Detection of autoactivation and toxicity of the bait protein expression plasmid CgTLR-TIR-BD. **(B)** Colonies on the selective medium SD/–Trp-Leu-His-Ade/Aba/X-α-Gal. **(C)** Testing of the blue colonies. M, DL2000 DNA marker; lines 1–113 show 113 blue positive colonies, which were picked and amplified by PCR using the T7/AD universal primers.

After the plasmid CgTLR-TIR-BD was successfully transformed into the Y2HGold strain, there were white colonies on the SD/–Trp medium, very pale blue colonies on the SD/–Trp/X-α-Gal medium, but no colonies on the SD/–Trp/AbA/X-α-Gal medium. Meanwhile, in the control group, white colonies appeared on both the SD/–Trp and SD/–Trp/X-α-Gal plates, and no colonies formed on the SD/–Trp/AbA/X-α-Gal plate (Figure 2A). These results suggested that CgTLR-TIR-BD could be expressed in the Y2HGold strain with no toxic effects. The fact that no colonies grew on the SD/–Trp/AbA/X-α-Gal medium indicated that CgTLR-TIR-BD could not activate GAL4-mediated transcription on its own.

During the mating experiments, the numbers of colonies on the selective medium SD/–Trp-Leu at 1/100 and 1/1,000 dilutions were 233 and 27, respectively. The average transformation efficiency and total number of colonies were 2.52 × 10^5^ cfu/μg and 2.52 × 10^6^ cfu. A total number of colonies >10^6^ indicates that the results of screening are reliable. Approximately 257 colonies grew on the SD/–Trp-Leu-His medium, and all of these were screened on the more selective medium SD/–Trp-Leu-His-Ade/Aba/X-α-Gal. After culturing at 30°C for 3 days, 113 colonies continued to grow and appeared blue to different degrees (Figure 2B). These clones were amplified with universal primers T7/AD, and results are shown in Figure 2C. The available PCR products were subsequently sequenced, leading to the identification of 27 different genes (Table 1). Three of these genes appeared at least four times in the screening and were related to MyD88.

**Table 1:** Putative genes identified in cDNA clones from the *C. gigas* cDNA library based on OysterBase—a database of oyster genes and omics.

**Clone No**.	**Best hit in the OysterBase**		**Frequency**
	**Gene_ID**	**mRNA**	
1	OYG_10020979	Myeloid differentiation factor 88-1 (Myd88-1)	5
2	OYG_10012725	PREDICTED: Myeloid differentiation primary response protein MyD88	9
3	OYG_10026092	Myeloid differentiation factor 88-2 (Myd88-2)	5
4	OYG_10026838	MAGUK p55 subfamily member 5 (MPP5)	2
5	OYG_10014743	Tripartite motif-containing protein 3 (TRIM3)	2
6	OYG_10008160	ATP synthase subunit beta-like	2
7	OYG_10024056	Arginine kinase (AK)	2
8	OYG_10011151	40S ribosomal protein S14	2
9	OYG_10022453	Histone deacetylase complex subunit SAP18	2
10	OYG_10009816	Annexin A7	2
11	OYG_10023712	Cleavage and polyadenylation specificity factor subunit 5	2
12	OYG_10026573	Oxoglutarate/iron-dependent oxygenase	2
13	OYG_10012428	NADH:ubiquinone dehydrogenase	2
14	OYG_10019936	Mitochondrial cytochrome c oxidase	2
15	OYG_10013347	ATP synthase subunit beta	1
16	OYG_10015851	Probable E3 ubiquitin-protein ligase	1
17	OYG_10006970	Cell division control protein 42	1
18	OYG_10014945	Complement C1q-like protein 4	1
19	OYG_10019358	Tripartite motif-containing protein 2	1
20	OYG_10009921	Coiled-coil domain-containing protein	1
21	OYG_10012610	Isocitrate dehydrogenase	1
22	OYG_10013041	Asparagine synthetase	1
23	OYG_10000050	Succinate dehydrogenase	1
24	OYG_10011616	Unknown proteins	3
25	OYG_10013773	Unknown proteins	3
26	OYG_10009179	Unknown proteins	1
27	OYG_10022667	Unknown proteins	1

**Figure 3:**
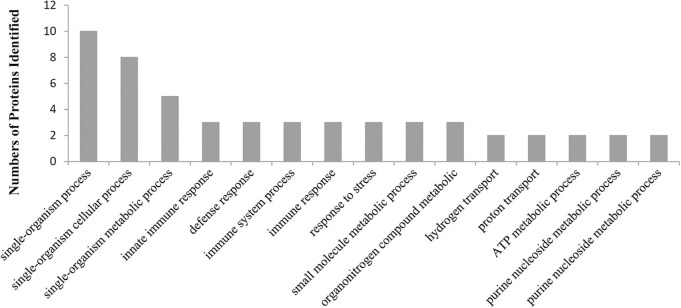
A graphic view of the distribution of CgTLR interacting proteins, separated into biological processes, as defined by the Blast2GO program (the top 15 of significant values, *p* < 0.05).

To gain some insight into the potential biological processes that are regulated by CgTLR protein, we classified all the identified host proteins using the Blast2GO program. For the proteins that bind with the CgTLR protein, the Blast2GO analysis identified 76 different biological processes defined in Table S2, as the function of a particular protein in the context of a larger network of proteins that interact to accomplish a process at the level of the cell or organism (Figure 3). Three proteins were classified as being involved in innate immune response and were related to MyD88; therefore, we focused on these proteins and further studied their functional relations.

### CgMyD88s Is a Homolog of MyD88 and Contains Only the TIR Domain

**Figure 4:**
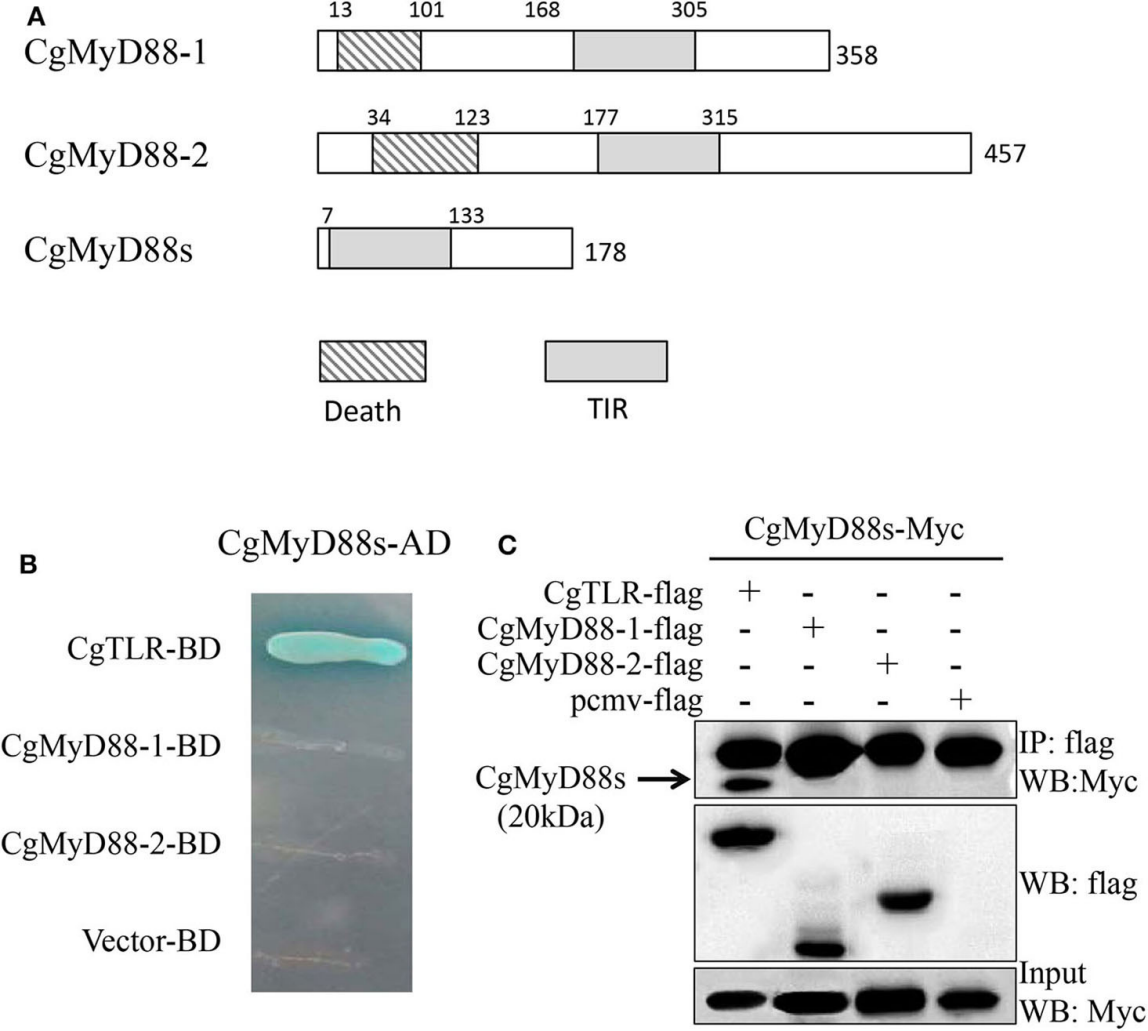
Interactions between CgMyD88s and CgTLR. **(A)** Schematic representation of CgMyD88-1, CgMyD88-2, and CgMyD88s protein. Death, death domain; TIR, toll/interleukin-1 receptor homology domain. **(B)** Determination of interactions using the yeast two-hybrid assay. The presence of blue clones on the quadruple dropout plate indicated direct interactions between CgMyD88s and CgTLR. **(C)** Determination of interactions using the co-IP assay. The band in the first line shows the interactions between CgMyD88s and CgTLR. The second and third lines were used to confirm expression of the co-transfected plasmids in the input sample.

MyD88-related genes in clones 1 and 3 have been characterized previously, whereas those in clone 2 have not (Table 1). After obtaining the full-length sequence of the predicted MyD88 primary response protein, we found that the CgMyD88s cDNA was 1,031 bp in length with a 537-bp ORF encoding a 178-amino-acid protein that was much shorter than that of CgMyD88-1 and CgMyD88-2. Beyond that, we found CgMyD88s contains only a TIR domain; it does not contain a death domain like that in the common MyD88 (Figure 4A). Therefore, we named it CgMyD88s, for “MyD88-shortened.” Meanwhile, based on a multiple amino acid sequence alignment analysis of the three oyster MyD88-like proteins with other MyD88 proteins, we found it was highly similar to the TIR domain of MyD88 (Figure S1).

### CgMyD88s Was Able to Interact With CgTLR but Not CgMyD88-1 or CgMyD88-2

To better understand the function of CgMyD88s in TLR-mediated signaling, we first investigated the relationship between CgMyD88s and CgMyD88-1, CgMyD88-2, and CgTLR. The Y2H system and co-IP were used to identify interactions between CgMyD88s and the other three proteins. For Y2H, blue colonies were obtained only with CgMyD88s and CgTLR together (Figure 4B). In the other groups, no colonies grew on the selective medium plates, indicating no interaction between the paired proteins. These results were confirmed by co-IP assay, which further showed that CgMyD88s was able to interact with CgTLR only (Figure 4C).

### OsHV-1 μVar Challenge Led to Lower Levels of CgMyD88s Gene Expression in Hemocytes

**Figure 5:**
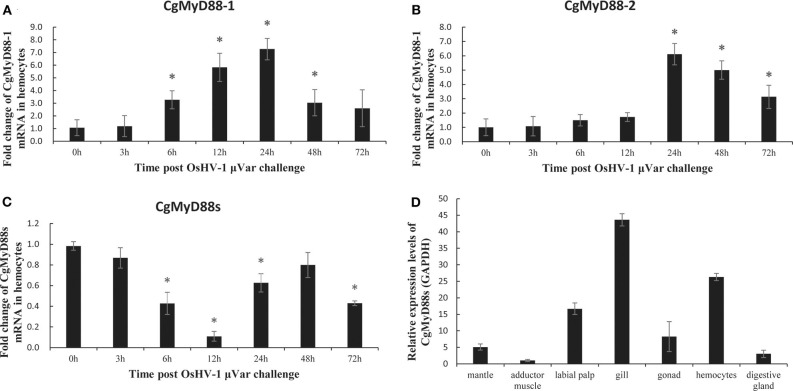
CgMyD88s expression in OsHV-1 μVar. mRNA expression profiles of CgMyD88-1 **(A)**, CgMyD88-2 **(B)**, and CgMyD88s **(C)** in hemocytes after OsHV-1 μVar injection. Expression patterns at 0, 3, 6, 12, 24, 48, and 72 h after treatment with OsHV-1 μVar. Results are displayed as fold changes from those in the control group. **(D)** Distribution of CgMyD88s expression levels in different tissues, normalized to levels of *GAPDH* expression. An adductor muscle sample was used as the reference sample. All of the results shown are from three independent experiments performed in triplicate. Data were presented as means ± SD. Asterisks above the bars indicate significant differences from the control groups (*p* < 0.05).

OsHV-1 μVar is a deadly oyster pathogen that stimulates the innate immune system of oysters. Analysis of mRNA expression after OsHV-1 μVar challenge revealed an obvious upregulation of CgMyD88-1 and CgMyD88-2 mRNA expressions (Figures 5A,B). In striking contrast to CgMyD88-1 and CgMyD88-2, the expression of CgMyD88s decreased in response to challenge (Figure 5C), suggesting that CgMyD88s may have a different function pattern than that of traditional MyD88. Similar to other immune-related genes in oysters, the tissue-specific expression of CgMyD88s was highest in hemocytes and gills and weak in adductor muscles (Figure 5D).

### CgMyD88s Competitively and Dose-Dependently Suppresses CgMyD88-Induced NF-κB Activation

**Figure 6:**
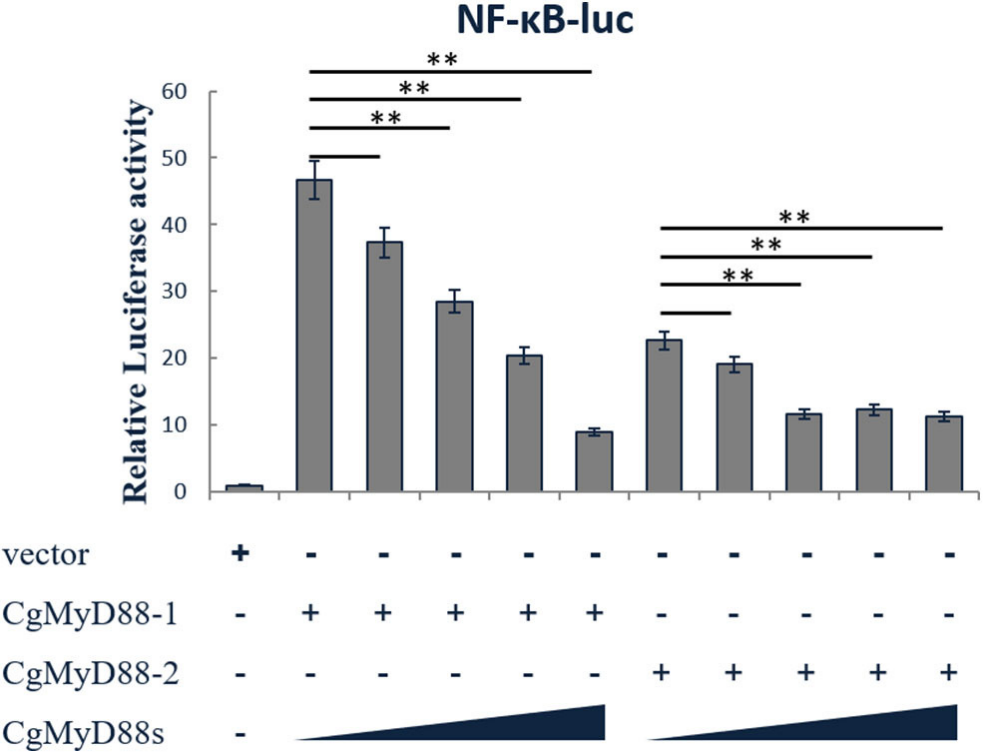
Dominant-negative effects of CgMyD88s in MyD88-induced NF-κB activation. HEK293 cells were co-transfected with increasing amounts of the MyD88s-Myc expression plasmid: 800 ng of the vector expressing CgMyD88-1-Myc or CgMyD88-2-Myc, 20 ng of pRL-TK as an internal reference, and 10 ng of the NF-κB-luc reporter plasmid. The data shown are means of three replicates after normalization for *Renilla* luciferase activity. The values are presented as means ± SD (*n* = 3), and significant (*p* < 0.01) differences from the CgMyD88-1-Myc or CgMyD88-2-Myc groups are indicated by **.

To study the activation of NF-κB, HEK293T cells were transiently transfected with an NF-κB-dependent luciferase reporter construct, CgMyD88-1 or CgMyD88-2, along with increasing amounts of CgMyD88s. Results showed that overexpression of CgMyD88-1 or CgMyD88-2 activated the NF-κB-dependent reporter construct, whereas, when CgMyD88s was added, the activation levels induced by CgMyD88-1 or CgMyD88-2 declined in a dose-dependent manner (Figure 6).

### Interference With CgMyD88s Expression Was Associated With Higher CgMyD88 Expression

**Figure 7:**
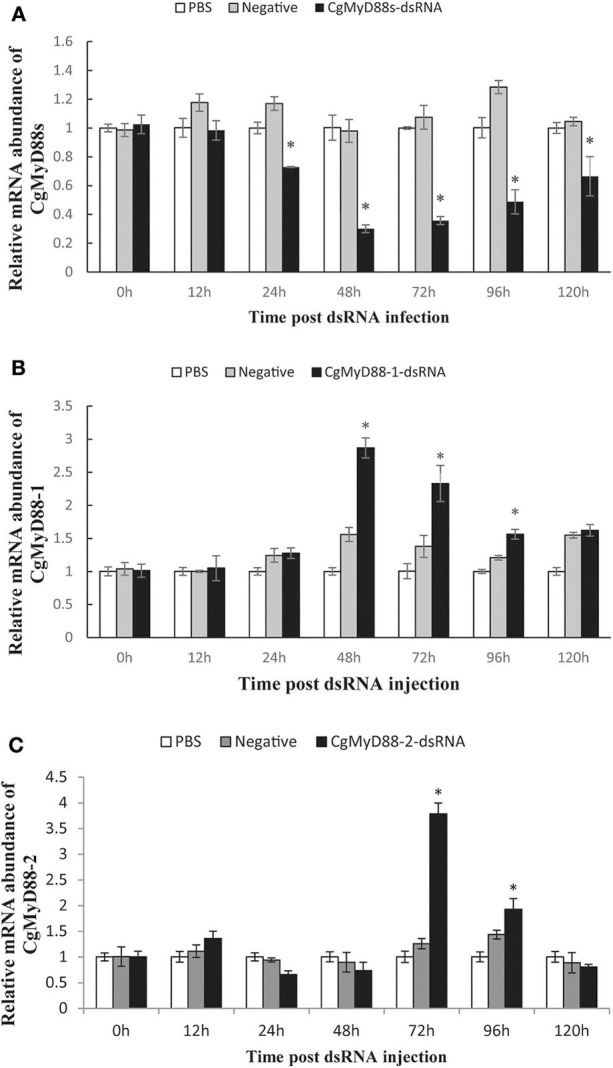
Expression profiles of CgMyD88-1 and CgMyD88-2 after interference with CgMyD88s expression. mRNA expression profiles of CgMyD88s **(A)**, CgMyD88-1 **(B)**, and CgMyD88-2 **(C)** in hemocytes after CgMyD88s interference. Expression patterns at 0, 12, 24, 48, 72, 96, and 120 h after treatment with PBS, EGFP, and CgMyD88s-dsRNA. Results are displayed as fold changes from those in the PBS group. All of the results shown are performed in triplicate. Data were presented as means ± SD. Asterisks above the bars indicate significant differences from the control groups (*p* < 0.05).

To better understand whether CgMyD88s is functionally related to CgMyD88-1 or CgMyD88-2, we silenced the expression of CgMyD88s through dsRNA interference and determined changes in expression of CgMyD88-1 and CgMyD88-2. We first tested whether the CgMyD88s TIR domain dsRNA could effectively silence the expression of CgMyD88s compared with empty vector and negative control groups (Figure 7A). As expected, we observed that slight inhibition occurred at 24 h (26% inhibition) and that expression was significantly inhibited at 48 and 72 h (70 and 66% inhibition, respectively).

Next, we determined the mRNA expression of CgMyD88-1 and CgMyD88-2 after effective interference of CgMyD88s. Results showed that expression of CgMyD88-1 was significantly upregulated beginning at 48 h after interference and then decreased slowly, whereas CgMyD88-2 expression was upregulated beginning at 72 h and then declined rapidly (Figures 7B,C). Both CgMyD88-1 and CgMyD88-2 were upregulated after effectively silencing the expression of CgMyD88s, suggesting that CgMyD88s may serve as a competitor of CgMyD88-1 and CgMyD88-2.

## Discussion

In this study, we identified 27 proteins that might interact with CgTLR. Three of these were MyD88-like proteins that likely serve as adaptors in TLR signaling pathways, whereas other annotated proteins may interact with CgTLR to participate in immune processes. Other unannotated sequences may have novel functions in TLR-mediated immune responses. Nevertheless, we focused on these three MyD88-like proteins as TLR-mediated innate immune adaptors. We identified, through Y2H screening, a new homolog of CgMyD88 named as CgMyD88s that interacted with CgTLR. Unlike traditional MyD88 (29, 30), CgMyD88s contained a C-terminal TIR domain but not an N-terminal death domain. The TIR domain was homologous to the TIR domain in Toll/IL-1R, which is a protein–protein interaction domain involved in hemophilic interactions with the TIR domains of TLRs (31). The death domain has been found in combination with various protein modules, including mammalian IRAK family proteins and *Drosophila* Pelle (32). Upon stimulation, MyD88 interacts with TLRs through the TIR domain and then recruits IRAK through interactions of the death domains of both molecules, which initiates innate immune signaling (33). CgMyD88s, however, only contains a TIR domain that might interact with TLRs but, because of the lack of a death domain, cannot transduce signals downstream. Based on the Pacific oyster genome and transcriptome, 10 MyD88-like genes have been identified; six possess a typical death–TIR domain combination and four possess only a TIR domain (20, 21). Of these, two MyD88-like genes correspond to the sequences we obtained from our Y2H screening (24).

Interestingly, although CgTLR, CgMyD88-1, CgMyD88-2, and CgMyD88s all have similar TIR domain structures, CgMyD88s interacted only with CgTLR and not CgMyD88-1 or CgMyD88-2. These results indicate that CgMyD88s and CgTLR cannot form a complex with CgMyD88-1 or CgMyD88-2. Furthermore, CgMyD88s might compete with CgMyD88-1 or CgMyD88-2 in their interactions with CgTLR.

Other TIR domain-containing proteins have been studied in mammals. For example, a TIR domain-containing adaptor protein (TIRAP) that does not contain an N-terminal death domain was found to function downstream of TLR4 through interactions with MyD88 upon recruitment to the receptor (34). The major difference between CgMyD88s and TIRAP are their interactions with MyD88. TRIF-related adaptor molecule (TRAM) is another adaptor that only contains a TIR domain (35). TRAM-deficient mice have defects in interferon-β production and activation signaling cascades mediated by TLR4, indicating that TRAM specifically participates in MyD88-independent TLR4 signaling pathways. In the absence of MyD88-independent pathway in invertebrates, CgMyD88s might serve a function different from that of TRAM.

TLR pathways are innate immune pathways: the expression of associated genes might increase or decrease in response to infection with certain pathogens. A previous study revealed that CgMyD88-1 and CgMyD88-2 transcripts were significantly upregulated in hemocytes after OsHV-1 challenge (24). In our study, we again demonstrated the upregulation of CgMyD88-1 and CgMyD88-2 expression in hemocytes after OsHV-1 μVar challenge. In addition, the expression of CgMyD88s was significantly decreased in the same conditions, revealing that CgMyD88s immune function was much different from that of CgMyD88-1 or CgMyD88-2. Although changes in CgMyD88s expression were very different from those of CgMyD88-1 and CgMyD88-2 after OsHV-1 μVar infection, the tissue distribution of CgMyD88s was similar to that of CgMyD88-1 and CgMyD88-2. All three of the genes encoding these proteins showed the highest expression in hemocytes and gills and were weakly expressed in adductor muscles (24). They all also interacted with CgTLR. These results indicate that CgMyD88s may be functionally associated with CgMyD88-1 and CgMyD88-2. Because we used recombinant plasmid to investigate protein–protein interactions *in vitro*, it is difficult to confirm that the protein is properly folded. Further study should use specific antibodies to identify protein–protein interactions *in vivo*.

Using dsRNA interference, we succeeded in silencing the expression of CgMyD88s. Along with decreasing the expression of CgMyD88s, interference may have caused CgTLR binding sites to open. CgMyD88-1 or CgMyD88-2 may have been rapidly upregulated to fill up these empty sites, which might explain why expression of CgMyD88-1 and CgMyD88-2 was upregulated with CgMyD88s interference. In addition, CgMyD88-1 expression was upregulated faster than that of CgMyD88-2 in the same conditions, which corresponded to changes observed after OsHV-1 μVar challenge. These results indicate that CgMyD88-1 may work faster than CgMyD88-2 to initiate an immune response.

To determine whether overexpression of CgMyD88s affects CgMyD88-1- or CgMyD88-2-induced immunity, we performed dual luciferase reporter experiments to identify changes in NF-κB activation. As expected, overexpression of CgMyD88s inhibited NF-κB activation induced by CgMyD88-1 or CgMyD88-2. This effect was more obvious with CgMyD88-1 than with CgMyD88-2. Previously, Xu et al. (36) identified two naturally truncated MyD88 variants in the Pacific oyster that also contained only TIR domains. Both of these variants were able to inhibit MyD88 activation of NF-κB activity. However, in the Xu study, these two variants were significantly upregulated in hemocytes after challenge with heat-killed *Listeria monocytogenes* (HKLM) and *Vibrio alginolyticus* (HKVA), a result which differed from that in our study.

**Figure 8:**
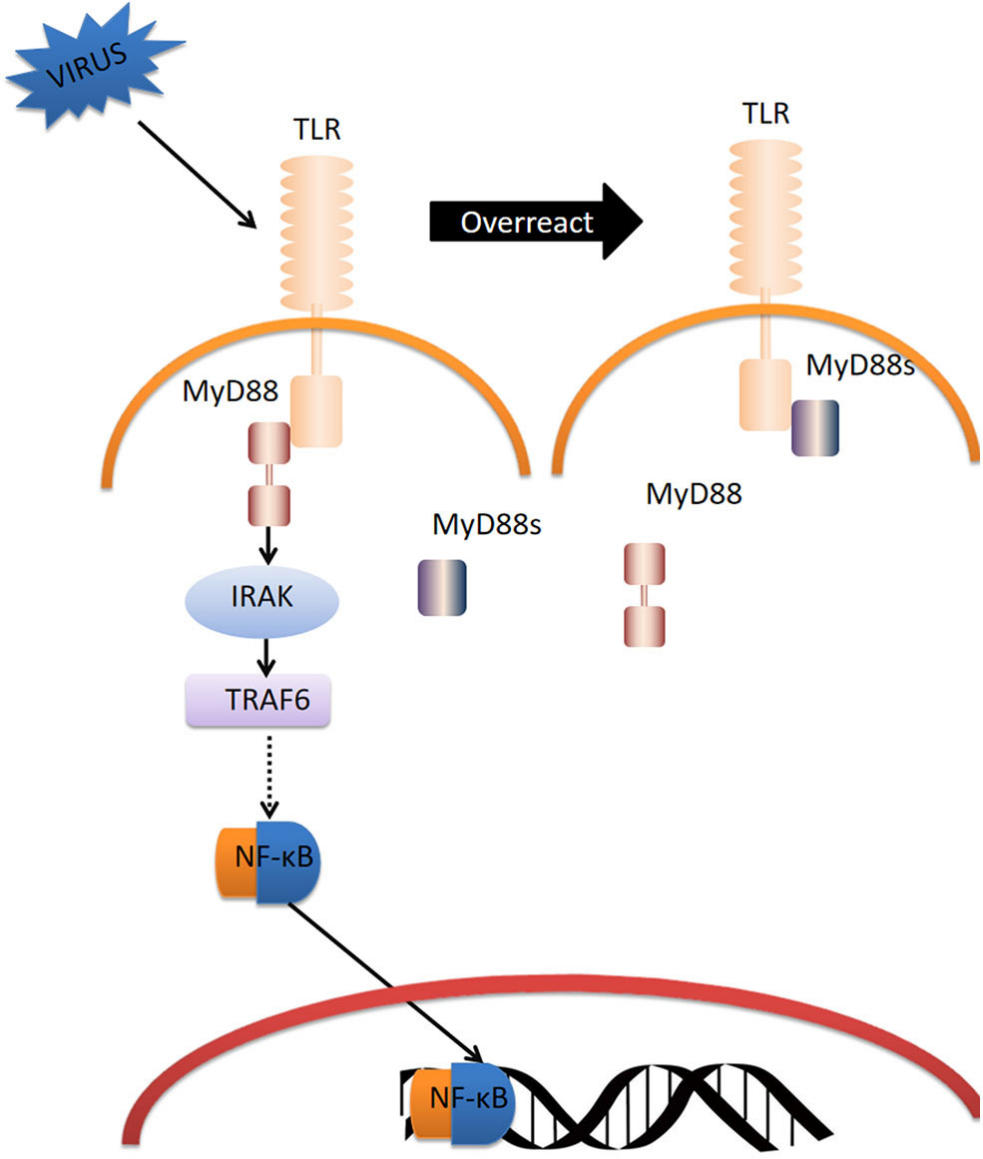
Model for the regulation of TLR signaling by CgMyD88s. Dotted arrow represents the abridged processes in TLR-MyD88 pathway.

In summary, we propose the following model of CgMyD88s function (Figure 8). CgTLR is activated by OsHV-1 μVar infection; CgMyD88-1 or CgMyD88-2 is recruited to bind to CgTLR and initiate an immune response. Once the host inflammatory response is too intense, CgMyD88s will edge out MyD88 and weaken the immunoreaction for maintaining oyster homeostasis. Thus, CgMyD88s may serve as a plug in the TLR-mediated innate immune system of the Pacific oyster, avoiding excessive inflammation.

Our novel findings might add evidence for the functional diversity of expanded immune genes in mollusks and other invertebrates. Zhang et al. (21) identified multiple innate immune gene families, including TLR pathway-related genes and RIG-I pathway-related genes, that exhibit significant expansion compared to genes in other model genomes. MyD88 coupled to TLR signaling via the TIR domain is extremely important in innate immunity and has undergone considerable expansion in oysters. The expansion of adaptors suggests a complex and divergent system of signal transduction. Mollusks have a very long evolutionary history of responding to variable environments that is embodied in genetic change through three main stages: origin through mutation, fixation, and preservation (37). The emergence of a new gene variant can result in gene copies, and functionally differentiated copies may be preserved in the evolution history of organisms (38). However, large numbers of innate immune-related receptors and adaptors likely have been lost during evolution, following the appearance of adaptive immunity. Our findings, however, might lead to new strategies to improve oysters' defenses against viruses by artificial initiation of innate immune pathways.

## Data Availability Statement

Publicly available datasets were analyzed in this study. This data can be found here: http://www.oysterdb.com/FrontHomeAction.do?method=home.

## Author Contributions

LL conceived and designed the study. XT and SL performed the experiments. WW assisted in collecting the samples. XT wrote the paper. BH, LL, and GZ reviewed and edited the manuscript. All authors read and approved the manuscript.

## Conflict of Interest

The authors declare that the research was conducted in the absence of any commercial or financial relationships that could be construed as a potential conflict of interest.
