# Supplementary material for: CgMyD88s Serves as an Innate Immune System Plug During Ostreid Herpesvirus 1 Infection in the Pacific Oyster (Crassostrea gigas)
Source: Front Immunol. 2020 Jul 14;11:1247. doi: 10.3389/fimmu.2020.01247 (PMC7381170; doi:10.3389/fimmu.2020.01247)
Supplement: Supplementary file 1 [file Table_1.DOCX]

**Table S1 Primer sequences and their use**

| **Primers** | **Sequences 5’→3’’** | **Reference** |
| --- | --- | --- |
| CgTLR-TIR-BD-F | CATGGAGGCCGAATTCCGAAGCAGAGCTGGTTATCA | Y2H^1^ screening system |
| CgTLR-TIR-BD-R | GCAGGTCGACGGATCCTTAAACAATGACCTCCCTCG |  |
| T7 | TAATACGACTCACTATAGGGC |  |
| AD | AGATGGTGCACGATGCACAG |  |
| C9 | GAGGGAAATTTGCGAGAGAA | virus quantitation |
| C10 | ATCACCGGCAGACGTAGG |  |
| CgMyD88s-RF1 | ACTTCACCCAGAACGAACAATG | Gene cloning |
| CgMyD88s-RF2 | ACCAGGTTTTCATTTGAGGCA |  |
| CgMyD88s-RR1 | GCCAGGACCGTCTCTTGATT |  |
| CgMyD88s-RR2 | CGTCGTAGCGGTTTATCTGAG |  |
| dTAP | GGCCACGCGTCGACTAGTACTTTTTTTTTTTTTTTT |  |
| dGAP | GGCCACGCGTCGACTAGTACGGGGGGGGGG |  |
| AP | GGCCACGCGTCGACTAGTAC |  |
| M13F | TGTAAAACGACGGCCAGT |  |
| M13R | CAGGAAACAGCTATGACC |  |
| CgMyD88s-IF | TAATACGACTCACTATAGGGTGTGATTGCGGGTGATAC | RNAi |
| CgMyD88s-IR | TAATACGACTCACTATAGGGATATCGGGCAAACAGACG |  |
| GFP-dsRNA-F | TAATACGACTCACTATAGGGAGTTCACCTTGATGCCGTTCTT |  |
| GFP-dsRNA-R | TAATACGACTCACTATAGGGCAGTGCTTCAGCCGCTACCC |  |
| CgMyD88-1-BD-F | CATGGAGGCCGAATTCCTTCACGCCCTTAACATG | Y2H |
| CgMyD88-1-BD-R | GCAGGTCGACGGATCCTTGGTCCTTGAGAAACACTA |  |
| CgMyD88-2-BD-F | CATGGAGGCCGAATTCCATTACGGGTTAGTGCAAGA |  |
| CgMyD88-2-BD-R | GCAGGTCGACGGATCCCCAGCTCGTCCACGTTAT |  |
| CgMyD88s-AD-F | TCCATCGAGCTCGAGCTATGGACAATCAGATAAACCAGTA |  |
| CgMyD88s-AD-R | CATCTGCAGCTCGAGCCTAATTGTTTCTCCACTCTTG |  |
| CgTLR-flag-F | GCTTCTGCAGGAATTCCGAAGCAGAGCTGGTTATCA | Co-IP^2^ |
| CgTLR-flag-R | CGACGATATCGAATTCTCATTAAACAATGACCTCCCTCG |  |
| CgMyD88-1-flag-F | GCTTCTGCAGGAATTCATGTCGATCACATCGGAACAG |  |
| CgMyD88-1-flag-R | CGACGATATCGAATTCTCAGCCGTTGTATGGAGTGTTGTC |  |
| CgMyD88-2-flag-F | GCTTCTGCAGGAATTC5ATGTCCATGTCGGAGATGGA |  |
| CgMyD88-2-flag-R | CGACGATATCGAATTCTCATCAGCAGTGGGAACTAGTGTTG |  |
| CgMyD88s-myc-F | CATGGAGGCCCGAATTATGGACAATCAGATAAACCAGTA |  |
| CgMyD88s-myc-R | CTCGGTCGACCGAATTCTAATTGTTTCTCCACTCTTG |  |
| CgMyD88-1-myc-F | CATGGAGGCCCGAATTATGTCGATCACATCGGAACA | DLR^3^ |
| CgMyD88-1-myc-R | CTCGGTCGACCGAATTTCAGCCGTTGTATGGAGTGT |  |
| CgMyD88-2-myc-F | CATGGAGGCCCGAATTTATGTCCATGTCGGAGATGGA |  |
| CgMyD88-2-myc-R | CTCGGTCGACCGAATTTCAGCAGTGGGAACTAGTGTTG |  |
| CgMyD88-1-qF | TTCAGACGAAAGGGCAATAGTGG | qRT-PCR^4^ |
| CgMyD88-1-qR | CATTCTACAAAACCTCGTGAATGAC |  |
| CgMyD88-2-qF | TGGAGTTGGGACGGTTGGAC |  |
| CgMyD88-2-qR | CACGTTATGATCGATGGCCGAC |  |
| CgMyD88s-qF | AGACTTCACCCAGAACGAACA |  |
| CgMyD88s-qR | ATGCAACTTGGTGCGAGACA |  |
| β-actin-F | GTGCTACGTTGCCCTGGACTT |  |
| β-actin-R | TCGCTCGTTGCCAATGGTGAT |  |
| GAPDH-F | TTCTCTTGCCCCTCTTGC |  |
| GAPDH-R | CGCCCAATCCTTGTTGCTT |  |

^1^ Y2H: Yeast-two-hybrid system

^2^ Co-IP: co-immunoprecipitation

^3^ DLR: Dual-Luciferase Reporter assay

^4^ qRT-PCR: quantitative Real-Time PCR
