# Supplementary material for: CgMyD88s Serves as an Innate Immune System Plug During Ostreid Herpesvirus 1 Infection in the Pacific Oyster (Crassostrea gigas)
Source: Front Immunol. 2020 Jul 14;11:1247. doi: 10.3389/fimmu.2020.01247 (PMC7381170; doi:10.3389/fimmu.2020.01247)
Supplement: Supplementary file 2 [file Table_2.DOCX]

Table S2. Biological processes of CgTLR-interacting proteins analyzed by Blast2GO.

| **GO.ID** | **Term** | **Significant** | **Pvalue** | **Genes** |
| --- | --- | --- | --- | --- |
| GO:0044699 | single-organism process | 10 | 0.01094 | CGI_10020979;CGI_10012725;CGI_10026092;CGI_10008160;CGI_10013347;CGI_10015851;CGI_10006970;CGI_10012610;CGI_10013041;CGI_10000050 |
| GO:0044763 | single-organism cellular process | 8 | 0.02264 | CGI_10020979;CGI_10012725;CGI_10026092;CGI_10008160;CGI_10013347;CGI_10015851;CGI_10006970;CGI_10013041 |
| GO:0044710 | single-organism metabolic process | 5 | 0.03542 | CGI_10008160;CGI_10013347;CGI_10012610;CGI_10013041;CGI_10000050 |
| GO:0045087 | innate immune response | 3 | 0.00046 | CGI_10020979;CGI_10012725;CGI_10026092 |
| GO:0006952 | defense response | 3 | 0.00067 | CGI_10020979;CGI_10012725;CGI_10026092 |
| GO:0002376 | immune system process | 3 | 0.00085 | CGI_10020979;CGI_10012725;CGI_10026092 |
| GO:0006955 | immune response | 3 | 0.00085 | CGI_10020979;CGI_10012725;CGI_10026092 |
| GO:0006950 | response to stress | 3 | 0.00998 | CGI_10020979;CGI_10012725;CGI_10026092 |
| GO:0044281 | small molecule metabolic process | 3 | 0.02316 | CGI_10008160;CGI_10013347;CGI_10013041 |
| GO:1901564 | organonitrogen compound metabolic | 3 | 0.02901 | CGI_10008160;CGI_10013347;CGI_10013041 |
| GO:0006818 | hydrogen transport | 2 | 0.00091 | CGI_10008160;CGI_10013347 |
| GO:0015992 | proton transport | 2 | 0.00091 | CGI_10008160;CGI_10013347 |
| GO:0046034 | ATP metabolic process | 2 | 0.00268 | CGI_10008160;CGI_10013347 |
| GO:0009126 | purine nucleoside metabolic process | 2 | 0.00373 | CGI_10008160;CGI_10013347 |
| GO:0009167 | purine nucleoside metabolic process | 2 | 0.00373 | CGI_10008160;CGI_10013347 |
| GO:0009161 | ribonucleoside monophosphate metabolic process | 2 | 0.00395 | CGI_10008160;CGI_10013347 |
| GO:0009123 | nucleoside monophosphate metabolic proce... | 2 | 0.00419 | CGI_10008160;CGI_10013347 |
| GO:0009144 | purine nucleoside triphosphate metabolic... | 2 | 0.00913 | CGI_10008160;CGI_10013347 |
| GO:0009199 | ribonucleoside triphosphate metabolic pr... | 2 | 0.00913 | CGI_10008160;CGI_10013347 |
| GO:0009205 | purine ribonucleoside triphosphate metab... | 2 | 0.00913 | CGI_10008160;CGI_10013347 |
| GO:0009141 | nucleoside triphosphate metabolic proces... | 2 | 0.00965 | CGI_10008160;CGI_10013347 |
| GO:0042278 | purine nucleoside metabolic process | 2 | 0.01055 | CGI_10008160;CGI_10013347 |
| GO:0046128 | purine ribonucleoside metabolic process | 2 | 0.01055 | CGI_10008160;CGI_10013347 |
| GO:0009119 | ribonucleoside metabolic process | 2 | 0.01129 | CGI_10008160;CGI_10013347 |
| GO:0009116 | nucleoside metabolic process | 2 | 0.01427 | CGI_10008160;CGI_10013347 |
| GO:1901657 | glycosyl compound metabolic process | 2 | 0.01427 | CGI_10008160;CGI_10013347 |
| GO:0009150 | purine ribonucleotide metabolic process | 2 | 0.01665 | CGI_10008160;CGI_10013347 |
| GO:0006163 | purine nucleotide metabolic process | 2 | 0.01688 | CGI_10008160;CGI_10013347 |
| GO:0009259 | ribonucleotide metabolic process | 2 | 0.0171 | CGI_10008160;CGI_10013347 |
| GO:0019693 | ribose phosphate metabolic process | 2 | 0.0171 | CGI_10008160;CGI_10013347 |
| GO:0072521 | purine-containing compound metabolic pro... | 2 | 0.01779 | CGI_10008160;CGI_10013347 |
| GO:0015672 | monovalent inorganic cation transport | 2 | 0.01968 | CGI_10008160;CGI_10013347 |
| GO:1901566 | organonitrogen compound biosynthetic pro... | 2 | 0.02343 | CGI_10013347;CGI_10013041 |
| GO:0009117 | nucleotide metabolic process | 2 | 0.03147 | CGI_10008160;CGI_10013347 |
| GO:0006753 | nucleoside phosphate metabolic process | 2 | 0.03386 | CGI_10008160;CGI_10013347 |
| GO:0055086 | nucleobase-containing small molecule met... | 2 | 0.04111 | CGI_10008160;CGI_10013347 |
| GO:0006812 | cation transport | 2 | 0.04343 | CGI_10008160;CGI_10013347 |
| GO:0006528 | asparagine metabolic process | 1 | 0.00288 | CGI_10013041 |
| GO:0006529 | asparagine biosynthetic process | 1 | 0.00288 | CGI_10013041 |
| GO:0009067 | aspartate family amino acid biosynthetic... | 1 | 0.00717 | CGI_10013041 |
| GO:0009066 | aspartate family amino acid metabolic pr... | 1 | 0.0086 | CGI_10013041 |
| GO:0043604 | amide biosynthetic process | 1 | 0.0086 | CGI_10013041 |
| GO:0007091 | metaphase/anaphase transition of mitotic... | 1 | 0.01003 | CGI_10015851 |
| GO:0030071 | regulation of mitotic metaphase/anaphase... | 1 | 0.01003 | CGI_10015851 |
| GO:0044770 | cell cycle phase transition | 1 | 0.01003 | CGI_10015851 |
| GO:0044772 | mitotic cell cycle phase transition | 1 | 0.01003 | CGI_10015851 |
| GO:0044784 | metaphase/anaphase transition of cell cy... | 1 | 0.01003 | CGI_10015851 |
| GO:1901987 | regulation of cell cycle phase transitio... | 1 | 0.01003 | CGI_10015851 |
| GO:1901990 | regulation of mitotic cell cycle phase t... | 1 | 0.01003 | CGI_10015851 |
| GO:1902099 | regulation of metaphase/anaphase transit... | 1 | 0.01003 | CGI_10015851 |
| GO:0007088 | regulation of mitosis | 1 | 0.01146 | CGI_10015851 |
| GO:0007346 | regulation of mitotic cell cycle | 1 | 0.01146 | CGI_10015851 |
| GO:0010564 | regulation of cell cycle process | 1 | 0.01146 | CGI_10015851 |
| GO:0051302 | regulation of cell division | 1 | 0.01146 | CGI_10015851 |
| GO:0051783 | regulation of nuclear division | 1 | 0.01146 | CGI_10015851 |
| GO:0051726 | regulation of cell cycle | 1 | 0.01856 | CGI_10015851 |
| GO:0043603 | cellular amide metabolic process | 1 | 0.01997 | CGI_10013041 |
| GO:0033043 | regulation of organelle organization | 1 | 0.02138 | CGI_10015851 |
| GO:0000278 | mitotic cell cycle | 1 | 0.0242 | CGI_10015851 |
| GO:0000280 | nuclear division | 1 | 0.0242 | CGI_10015851 |
| GO:0007067 | mitotic nuclear division | 1 | 0.0242 | CGI_10015851 |
| GO:0048285 | organelle fission | 1 | 0.0242 | CGI_10015851 |
| GO:1901607 | alpha-amino acid biosynthetic process | 1 | 0.0242 | CGI_10013041 |
| GO:1903047 | mitotic cell cycle process | 1 | 0.0242 | CGI_10015851 |
| GO:0051301 | cell division | 1 | 0.02701 | CGI_10015851 |
| GO:0008652 | cellular amino acid biosynthetic process | 1 | 0.03122 | CGI_10013041 |
| GO:0022402 | cell cycle process | 1 | 0.03262 | CGI_10015851 |
| GO:0051128 | regulation of cellular component organiz... | 1 | 0.03262 | CGI_10015851 |
| GO:0015985 | energy coupled proton transport, down el... | 1 | 0.04097 | CGI_10013347 |
| GO:0015986 | ATP synthesis coupled proton transport | 1 | 0.04097 | CGI_10013347 |
| GO:0034220 | ion transmembrane transport | 1 | 0.04235 | CGI_10013347 |
| GO:0098655 | cation transmembrane transport | 1 | 0.04235 | CGI_10013347 |
| GO:0098660 | inorganic ion transmembrane transport | 1 | 0.04235 | CGI_10013347 |
| GO:0098662 | inorganic cation transmembrane transport | 1 | 0.04235 | CGI_10013347 |
| GO:1902600 | hydrogen ion transmembrane transport | 1 | 0.04235 | CGI_10013347 |
