# Supplementary figures and images for: CgMyD88s Serves as an Innate Immune System Plug During Ostreid Herpesvirus 1 Infection in the Pacific Oyster (Crassostrea gigas)
Source: Front Immunol. 2020 Jul 14;11:1247. doi: 10.3389/fimmu.2020.01247 (PMC7381170; doi:10.3389/fimmu.2020.01247)

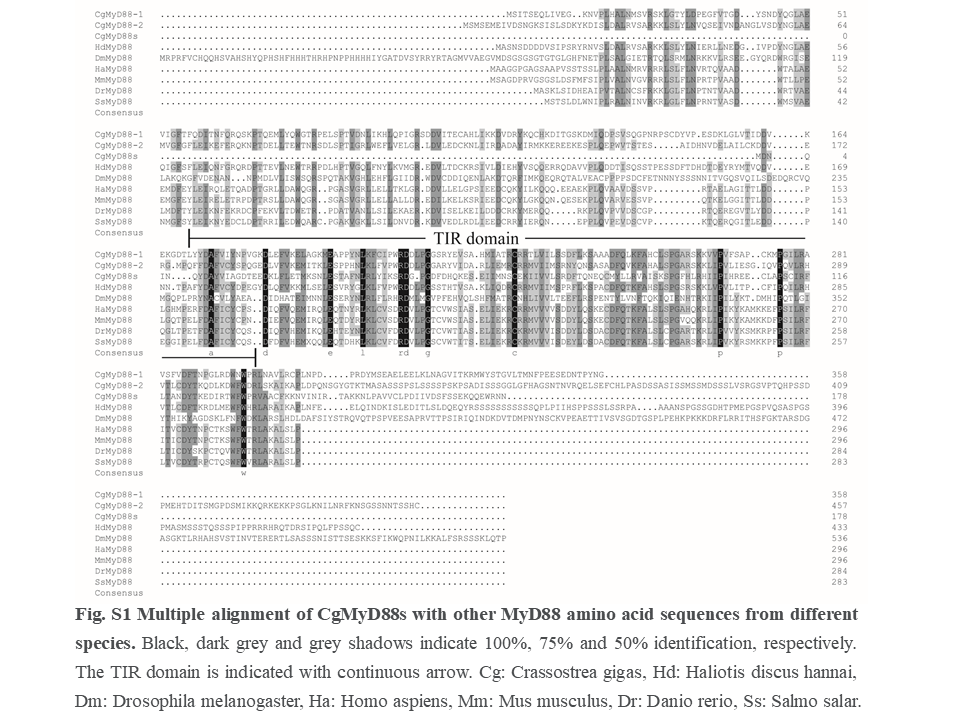

Supplement: Supplementary file 4 [file Image_1.TIF]
